# Miscellaneous Notices

**Published:** 1855-07

**Authors:** 


### EDITORIAL DEPARTMENT.

### MISCELLANEOUS NOTICES.

*Drs. Allen and Hunter.*—The suit brought by Dr. Allen more than a year ago, against Dr. Hunter, for an infringement of a patent obtained by him for mounting artificial teeth with a fusible continuous gum, having elicited considerable interest throughout the dental profession we give place, to the exclusion of other matter designed for this part of the Journal, to the following brief synopsis of the trial. By this, it seems that neither the validity of the patent has been invalidated, nor an infringement on the part of Dr. Hunter established. If the trial has been productive of no other consequences, it has at least put an end to the bitter controversy which had been carried on for some years by the two parties, and we are gratified to learn, has resulted in a reconciliation.

The improvement introduced and perfected by these gentlemen, in the method of mounting artificial teeth, is certainly the most valuable of any ever made in dental prosthesis, and will very soon be universally adopted. Both deserve great credit for their ingenuity and perseverence, and we would like to see some expression of the appreciation of the profession of their improvement.—Eds.

A Synopsis of the late trial in the United States Circuit Court for the Southern District of Ohio, in the case of J. Allen *vs.* Wm. M. Hunter.

This important case, says the Cincinnati Gazette, "involving a patent for one of the most valuable improvements known in the dental art, 'a new mode of setting artificial teeth on metallic plates, secured by John Allen, of Cincinnati, in December, 1851,' which Dr. W. M. Hunter, also of our city, was alleged to have infringed, came to a conclusion on Saturday evening, after a long and able closing argument by Henry Stanbery, and charge of Judge McLean. This trial occupied eight days, during which time fifty-seven depositions were read, and thirty-one persons examined before the jury. There were ten of the depositions, and five of the witnesses for the plaintiff in opening the case; eighteen of the depositions and nineteen of the witnesses were for the defence. The plaintiff as rebutting evidence, read twenty-nine depositions and examined seven witnesses. The testimony in this case was from men of the highest character in the dental and chemical professions, of our own city, and from various cities in the United States and in Europe. Great interest was felt in this case in both countries, as letters patent had been secured in each by the plaintiff for this improvement. There were two points involved, the validity of the patent and the infringement by the defendant. The patent was sustained, but the point of infringement was not. The plaintiff, it seems, had relied upon testimony showing that the defendant had sold formulas and given instructions to others for producing the work, but there was no law against selling formulas or giving instructions, as those only were responsible for damages who reproduced substantially the thing patented. The plaintiff produced in evidence but one piece of work, made after his plan by defendant, prior to the commencement of the suit, and that was for an old lady for which he had made no charge, consequently no damages were shown, and the jury returned a verdict of not guilty of infringement. Again, it was shown, that Drs. Allen and Hunter had each been prosecuting a series of experiments, which, although widely different, in the beginning, yet, both finally reached the same practical result.

The defendant plead the general issue which involved the questions of priority, sufficiency of specifications, utility, &c. As to priority, the plaintiff showed, by numerous witnesses, that he had been prosecuting his experiments for a number of years to get a compound that would unite single teeth to each other, and to the plate upon which they were set, so as to form a continuous artificial gum. Dr. Geo. L. Weed testified that he lived second door to Dr. Allen, and knew that he had been experimenting for at least ten years, to accomplish what he had at length attained, that he often saw him working at it, very late at night with untiring zeal to accomplish his object, had seen specimens of work, from time to time, until the date of his patent, and that he had tested the work with a full set made for his wife, and considers it incomparably superior to the old method.

Dr. A. Curtis, of Cincinnati, deposed, that he had known Dr. Allen to have been working at this improvement for some ten years; had often been in his laboratory during that time, and had seen specimens of his work, with an artificial gum flowed upon the teeth and plate. Dr. J. McCullum, of Augusta, Ky., had seen work of this kind done by Dr. Allen in 1846.

Mrs. Colonel Bartlet, of Covington, Ky., testified, she had a full set of teeth made by Dr. Allen with continuous gums fused upon the teeth and plate in 1846 or 7, though not as perfect as a subsequent set made upon the same plan, which were exhibited in court, and appeared as perfect as when first made, had worn the old style but considered the new far superior. Numerous other witnesses corroborated the foregoing testimony on those points. It was urged on the part of the defence, that substantially the same thing had been done before by Delabarre of Paris, many years ago, and that it had been published in Fitch's Dental Surgery.

Dr. S. S. Fitch, plaintiff's witness, testified that he was the author of the above named work, and that he *knew* Delabarre had never perfected his experiments or brought them into practical use, that he (Fitch) had spent over one thousand dollars, and one year's time in vain attempts to produce a practical result, but never could obtain it from Delabarre's formulas, nor did he ever know of any one who could, although he was well acquainted in Paris, had spent much time there, and knew well the French mode of practice. That he had seen Dr. Allen's continuous gum work, and considered it far in advance of any thing he had ever before witnessed in the dental line.

Professor Silliman, (senior,) of Newhaven, testified that he had never seen any work of this kind until it was introduced by Dr. Allen; that he had tested it in his family for some three years, and considered it "*preeminently*" useful.

Dr. E. Slack, of Cincinnati, stated, he was the oldest professor of chemistry in the United States except Prof. Silliman. That he, in connection with Dr. M. W. S. Kendal, tested the two formulas of Allen and Hunter, by actual experiment in the laboratory of the Ohio Dental College, that he considered them substantially the same.

Dr. J. Lock, Jr., chemist, of Cincinnati, deposed, that he had with great care produced a chemical analysis of the ingredients named in the formulas of Delabarre, Allen and Hunter, that Delabarre's compound required thirty-seven hundred and sixty-one degrees of heat to fuse it. This was owing to a great excess of alumina and the want of a sufficiency of fusible materials to unite the particles firmly together; consequently he found that Delabarre's compound when fused, was two fragile for practical purposes in the mouth, while Allen and Hunter's fused at two thousand two hundred and four degrees of heat possessing a much larger proportion of fusible materials, and producing good practical results, as shown by experiments conducted by himself, assisted by Dr. Kendal at the instance of the plaintiff, in the Dental College laboratory.

The report of Dr. Lock, is a very full and clear one, containing much valuable information with reference to the nature and physical results of various mineral compounds. This report will be furnished in the next number of the Journal.

Dr. James Robinson, of London, author of Robinson's Work on the Teeth, testified that he had never seen any work of this kind, until he saw Dr. Allen's; that he had become acquainted with it through his agent in London, that he regarded it new and useful.

Dr. Jobson, author of Jobson's Treatise on the Teeth, testified that he was well acquainted with Delabarre, and knew that he had not brought his method into practical use, that he had abandoned his experiments long since; that Delabarre had frequently shown him the results which he had obtained, but they were not such as he had seen produced by Dr. Allen, that he had never seen anything of the kind to compare with the work he had seen done by the patentee, that it is new and useful, and left little or nothing more to be accomplished in the construction of artificial dentistry. Dr. J. S. Clark, of New Orleans, testified that Delabarre had never perfected his plan of forming a continuous artificial gum, so as to be rendered practical; that he was acquainted with Dr. Allen's method, had tested it some two years or more, considered it new and very useful, and would not be without it for two thousand dollars, that he had a large and full practice, the largest in New Orleans. Dr. James Taylor, Professor in the Ohio College of Dental Surgery, stated that Delabarre's method was never perfected, or brought into practical use, that he considered Dr. Allen's new and useful; had never seen anything of the kind prior to his.

Dr. E. Baker, of New York city, had been in practice some forty years, but never saw any work of this kind prior to its introduction by Dr. Allen, was in the way of knowing it, if anything of the sort had been previously in use.

Dr. J. C. Colburn, of Newark, New Jersey, had adopted this method exclusively in his practice, during the last year; never saw anything, or any work like it prior to its introduction by Dr. A. Thinks it far superior to any other method. Dr. S. P. Miller, of Worcester, Mass., testified he had bought the right to use this method in his practice, had inserted between two and three hundred sets within the last three years, which have given entire satisfaction, that he formerly made blockwork, but thinks Allen's method far superior to any other, with which he was acquainted. Dr. J. Simmonds, of Providence, R. I., testified that he, in conjunction with Dr. Haws, had constructed between four and five hundred sets upon Dr. Allen's plan, which have given general satisfaction, that this style of work is generally preferred in that vicinity to all others, that they formerly made block work, but prefer the continuous gum. There were, also, many other witnesses who testified that they considered this method new and far superior to any other. There were others who were not accustomed to block or furnace work, who had not succeeded so well, either from want of experience or confidence to persevere. As further evidence, this style of work had been made before defendant exhibited a dilapidated upper denture, accompanied with the deposition of Dr. Smily, of New York. This piece was said to have been worn by Aaron Burr, but who made it, when or where it was made, did not appear in evidence. The material of which it was made, seemed very different from Allen's or Hunter's. It was also urged by defendant in his plea, that Dr. George Haws, of New York, had seen this kind of work before its introduction by Dr. Allen. Dr. Haws testified that the specimens he saw were made by Dr. Ambler, of New York. Dr. Ambler testified that the efforts he made rested in experiment, and that he never brought it into practical use, that he did not consider what he did as identical with Dr. Allen's, that it was very different from his. It was, also, set forth in defendant's plea, that Morris Levett, of New York, had also produced the same result, before the date of Allen's patent. Dr. Levett testified that what he had produced was entirely different in the nature of the compound, the object to be attained, and in the practical results. Other witnesses also testified to having made experiments with a view to accomplish a similar result, but had not reached the same practical point as Drs. Allen or Hunter. Dr. Harrison, of Buffalo, testified he had united teeth to each other, thus forming a block without any plate, a specimen of which was exhibited by Dr. Bristol, of Lockport, New York, who had also assisted Dr. Harrison in his experiments. Drs. Bears and Morgan, of Rochester, New York, had fused an enamel upon a set of teeth, mounted upon gold plate, for a Mr. Wilson of New York, but which did not stand the saliva of the mouth, and was considered by witnesses, Holden and Putnam, who examined the work, to be unfit for dental purposes.

It was shown on the part of the defence, that the defendant had produced good practical results in the early stages of this improvement.

Thus it would seem from the result of this investigation, that although the profession have long felt the necessity for something of this kind, and experiments have been made from time to time, both in this country and in Europe, for the last thirty years, to accomplish this desirable object, yet to the parties in this suit must be awarded the credit of having attained it. Since the termination of this suit, an amicable adjustment of all differences between the parties has been made.

This is highly gratifying to the friends of both parties, for controversies of this kind are attended with great expense and anxiety of mind.

The patent continues valid in the hands of the patentee.

*Contribution to the Museum of the Baltimore College of Dental Surgery.*—Dr. Maynard of Washington city, recently presented to the Museum of this institution, his extensive collection of maxillæ, embracing some two hundred specimens, beautifully prepared with a view to demonstrate the maxillary antra, showing their great variety of conformation and size as well as the relationship which they sustain to the teeth and many of the morbid changes to which they are subject. This, no doubt, is the most complete collection in the world, and their value is very greatly enhanced by the beautiful manner in which they are prepared. It constitutes a most valuable addition to the means of instruction in the Baltimore College, and the faculty are under great obligations to Dr. M. for so valuable a donation.

---

*Correspondence.*—It gives us pleasure to insert the following correspondence. Our regret that any misunderstanding should have occurred is overcome by our gratification at the desire shown by both Schools to preserve unbroken their amicable relations, and to allow no disreputable contentions to interfere with the progress of Dental Education.

*Baltimore, January 19th,* 1855.

Dr. Robert Arthur,

*Dean of the Philadelphia College of Dental Surgery.*

Dear Sir:—With deep regret the Faculty of the Baltimore Dental College learn the construction put by yourself and colleagues upon a circular sheet, issued in February last, for distribution among members of the medical profession.

I quote the following clause: "The Faculty desire to direct attention to the wording and emphasis of the above conditions, having noticed with regret a tendency on the part of some to lower the standard of professional qualification. Such a step might give a temporary increase to the number of their students: but looking beyond the present, and influenced by other considerations than mere pecuniary interest, it is their determination in no case, by abating aught from their measure of requisite preparation, to endanger the honor of the profession, or the present high position of their school."

The general tenor of the circular, you say, warrants the inference from these words, that we had noticed and felt aggrieved by conduct on the part of institutions similar to our own, calculated "to lower the standard of professional qualification" for "pecuniary" gain. This is the construction put upon them by "every intelligent man in and out of the profession," with whom you have conversed, and "is the only sense in which these words can be read by those who have not the advantage of your [my] own exposition of them.

Justice to my own colleagues, equally as to yours, demands that, as the author of that circular, I should state for whom, and for whom alone, the words thus misunderstood were intended. In giving you the liberty to make public my "exposition, I offer all the reparation in my power for an unintended injury.

Frequent letters have been addressed to me, as Dean, requesting graduation upon easier terms than those set forth in our circular, and coupling this request with plain intimations that a much less degree of preparation would suffice for the dentist. Especially prevalent in the kindred profession of medicine is this depreciating estimate of the standard of dental qualification. These correspondents were the "some" referred to in the above quoted paragraph, these and such like were those who, upon our acceptance of their terms, might add to the number of our students, and the amount of our fees, to the permanent injury both of our school and profession. Any words in the circular that could suggest a meaning beyond this, I must regard as most unfortunately chosen. I certainly hold myself incapable of any species of covert insinuation.

Deprecating as I do all contentions, individual or corporate, and most reluctant to mar the youth of dental instruction with painful jealousies, such as stain the riper years of medical education, I would never widen an existing breach, far less be the first to throw the apple of discord.

In heartily wishing the schools of Philadelphia and Cincinnati success in their work of professional advancement, we indulge no affectation of disinterestedness. If only one is to succeed at the expense of the rest, we would have ours the successful one in an experiment, which it was the first to try. But dental collegiate education is so rapidly becoming a universally acknowledged necessity, that there is ample room for all to labor; not as jealous rivals, but as friendly co-workers in a new cause. The success of each, by giving an increased impetus to this common cause, benefits the others, and in this reciprocity lies the secret, "Strength of Union," which I should be loth to see weakened by dissentions.

We shall ever labor to increase the prosperity of our school and to deserve it, but it forms no part of these labors to depreciate the efforts of others. We sincerely wish you continued success, and earnestly hope that with this, now I trust, explained misunderstanding, may pass away every unpleasant feeling calculated to mar the harmony between us.

Very respectfully, yours,

P. H. AUSTEN, *Dean of the Faculty, B. C. D. S.*

*Philadelphia, June 26th,* 1855.

Dr. P. H. Austen,

*Dean of the Baltimore College of Dental Surgery.*

Dear Sir :—Your communication of the 19th inst. was duly received. It cannot be otherwise than satisfactory in all respects to the Faculty of the Philadelphia College, explaining as it does the true meaning of the circular alluded to. Misunderstandings may arise between those who are best disposed towards each other, but they are never difficult to settle when both parties are actuated by right purposes. It affords me pleasure to find you so ready to take the proper steps to remove an impression, which we and others have regarded as capable of doing us injury.

You may rest well assured that you cannot be more anxious to avoid conflict than are those connected with the Philadelphia College. We have engaged in the work of instruction in our profession with a true and earnest purpose, and we have from the beginning, found our hands too full of our legitimate duties to allow ourselves to be influenced by any petty spirit of rivalry towards other schools.

You may rest well satisfied that the Baltimore school has the best wishes of the Faculty I represent, for its continued success, so long as it is conducted with a due regard to the interests of the profession and the public, and you will certainly always find in us, instead of "jealous rivals,"

hearty and most friendly co-workers in the good cause in which we have engaged. With sentiments of esteem for yourself and colleagues, I remain,

Very respectfully, yours,

R. ARTHUR,

*Dean of the Faculty of the P. C. D. S.*

---

Messrs. Editors—Within I send you the enclosed letter for publication in your Journal, as the best method of making known to the profession the act of generosity therein contained. Your ob't serv't,

P. H. AUSTEN, *Dean B. C. D. S.*

*To the College of Dental Surgeons, Baltimore, Md.*

Gentlemen—In the year 1847, I filed my caveat, and in 1848 I obtained a patent for my invention of an instrument of dental surgery, called "the Compound Union Screw Forceps,"* which I have found of singular use in the extraction of teeth, which, from external defects or caries are liable to be crushed by the pressure of the forceps in common use. I have been highly gratified that some of the most accomplished dentists, particularly in the Southern states, have expressed their admiration of the instrument, and testify to its great value and utility in delicate cases.

My sole object in obtaining the patent was to establish my right to the invention. It has been a source of some revenue to me, which I ever regarded as of far less consideration than the vindication of my exclusive right to, and prior invention of, the improvement and the principle of its action. Having vindicated these, I am now desirous of presenting the invention to the free use of the dental profession throughout the United States, and could think of no corporate body connected with our science commanding so high a position in the estimation of the world as your college. I, therefore, most respectfully present the patent to your free use, in testimony of the high regard I entertain for your sound erudition, and the wide spread usefulness that has emanated from your institution.

My agents, H. G. Kern, of Philadelphia; J. M. Brown, of Cincinnati, and T. M. Brown, of Louisville, from the date of the publication of this, will be held under no responsibility to me on account of said patent.

Very respectfully, gentlemen,

Your most obedient servant,

CHARLES H. DUBS, *Dental Surgeon.*

Natchez, *Feb'ry* 3*d*, 1855.

---

*Warranted.*—This is a word, we regret to say, too often seen in the advertisements of dentists, especially among those whose claims to scientific attainments and skill are not of the most elevated character. It has always seemed to us as a sort of acknowledgment of want of merit, and held out as an inducement to the public to employ the advertiser. It appears to be one of the thousand artifices resorted to by psuedo dentists, to obtain prac-

* A description of an instrument similar to this, accompanied by an engraving, invented by Dr. S. P. Hullihen, was published in the Journal some six or seven years ago. The only difference is, that the screw of Dr. H's is moved by means of a spiral spring and Dr. D's by a ratchet.

tice. We cannot believe that a man conscious of ability, capable of holding out an inducement so humiliating and degrading to professional dignity, to render his services marketable. "All operations warranted;" how absurd the idea! If a dentist makes an operation as perfect as the circumstances of the case will admit, if it fails, it is no fault of his, and should he be called upon to reperform it, he is as justly entitled to compensation for the last as for the first operation. The failure being the result of the peculiar circumstances of the case, not want of skill on his part, the patient can have no claims whatever upon him, and should he employ him a second time, it should be with the full intention of paying him a fair price for his services, that being all that was charged in the first instance. A dentist might with just as much propriety be expected, when he fills a tooth under circumstances which render the success of the operation at all doubtful, to do it gratuitously in the first as in the second instance. The patient, in a case of this kind, desiring to have the operation performed, and knowing the difficulty of the case, must take the risk.

But a dentist conscious of inability to exercise his professional duties in a skillful manner, and knowing or suspecting that others are aware of the fact, might, we admit, with very great propriety, hold out a ***warrantee*** as an inducement to the public to employ him.

The above remarks were elicited by the receipt of a letter from a professional friend, as our last sheet was going to press, asking our opinion with regard to the propriety of dentists warranting their operations. At some future time, when we have more leisure and space, we may present our views upon the subject at greater length.

*The Chemico-Physiological Effects of Coffee.*—The following abridgment of the translation of a paper by Julius Lehmann, on the above subject, published originally in the Annalen der Chemie and Pharmacie, was designed for the Quarterly Summary, but being too important to defer, and having already lain over twice, we have concluded to incorporate it in the Editorial Department.

Lehmann took two healthy men as the subjects of his experiments. He put them on a plain and not very full diet, but sufficient to satisfy them, and better than they had been accustomed, coffee being excluded. At first they suffered from feelings of depression and debility, and passed turbid alkaline urine. After a time, however, their unpleasant symptoms wore off, and the urine became regular in quantity and in constitution. At this time it was ascertained that these two men, G. M. and H. S., voided daily:

| G. M. | H. S. | |
|---|---|---|
| 1444 | 1635 | cc. of urine, containing |
| 4.140 | 4.421 | grammes of phosphoric acid, |
| 9.363 | 9.865 | " " chloride of sodium, |
| 27.232 | 31.298 | " " urea. |

This being ascertained to be the average daily excretion, by careful analysis, extending over a fortnight, it was next determined to ascertain what effect coffee would produce. Accordingly the men were continued on the same diet, with this exception, that they took coffee at breakfast and dinner, each portion prepared from ¾ oz. of commercial coffee. From the first day of this diet, their depression and languor passed off, and they became cheerful and well disposed to work. The urine remained entirely normal.

There was a marked decrease in the solid constituents of the urine, as will be seen by comparing the following table with the preceding. They passed:

| G. M. | H. S. | |
|---|---|---|
| 1512 | 2005 | cc. of urine, containing |
| 3.105 | 3.001 | grammes of phosphoric acid, |
| 6.951 | 8.819 | " " chloride of sodium, |
| 20.695 | 21.888 | " " urea. |

Coffee is thus seen to retard the metamorphoses of the tissues.

They were now put upon a decoction of 1½ oz. of coffee, whereupon appeared a greatly increased action of the heart, movement of the bowels, perspirations, anxiety and giddiness, which were followed by weakness and sleep broken through unpleasant and confused dreams.

The question now arose: upon what do these effects depend? To determine this point, G. M. received caffeine, morning and noon, in place of his coffee. When his coffee was discontinued and he returned to his former usual diet, he suffered from the depression, which passed off as soon as the caffeine was commenced.

The daily average was:

Urine, 1928 cc.; phosphoric acid, 3.768; chloride of sodium, 9.546; urea, 24.088. The decrease in the solids was not so marked as after using coffee.

After taking 8 grains of caffeine daily, he had quickened pulse, strong action of the heart, trembling and continued desire to urinate, but with the ability to pass but a small quantity.

To establish this point he gave caffeine to another person, with the following result:

*Diet without Caffeine.*—Urine, 1200 cc.; chloride of sodium, 7.790; phosphoric acid, 3.910; urea, 25.150.

*Diet with Caffeine.*—Urine, 1340 cc.; chloride of sodium, 6.890; phosphoric acid, 3.705; urea, 22.230.

The investigation was then delayed eight days, and he began again:

*Diet without Caffeine.*—Urine, 1250 cc.; chloride of sodium, 6.980; phosphoric acid, 3.855; urea, 25.010.

*Diet with Caffeine.*—Urine, 1276 cc.; chloride of sodium, 6.790; phosphoric acid, 3.690; urea, 20.800.

Caffeine, therefore, retards metamorphosis.

The next investigation related to the action of the empyreumatic oil. To obtain this, roasted coffee was distilled with water till the residue was inodorous. G. M. took daily four glasses of this, which contained the empyreumatic oil of two ounces of roasted coffee.

The average per day was:

Urine, 1789; phosphoric acid, 3.479; chloride of sodium, 10.307; urea, 20.271.

This produced pleasant excitement and gentle perspiration, and the depressed feeling passed away as before. The watery contents of the urine increased, while the phosphoric acid and the urea diminished. The oil, it will be observed, retards metamorphosis more than the caffeine. A double dose produced congestion, profuse perspiration and sleeplessness. In two other cases it produced diarrhea.

Lehmann sums up his results as follows:

1. Coffee stimulates the vascular and nervous systems, while at the same time it retards metamorphosis.

2. The stimulating effect is due to the opposing modifications of the action of the empyreumatic oil and the caffeine.

3. The retardation of metamorphosis is due mainly to the oil.

4. Increased activity of the heart, trembling, dizziness, &c., are produced by caffeine.

5. The increased perspiration, nicturition and peristaltic action are due to the oil.

Then follows an examination of the various methods of using coffee and tea, as practiced by different nations. The conclusion he comes to is that they are valuable as economizing nutrition. While they retard metamorphosis, they diminish the necessity for nutrition, and thus are cheap indirect nutriments. Coffee influences more directly the metamorphosis, and tea the nervous system. The comments upon the national characteristics of the use of these articles, and their influence upon life, are very interesting, but we regret that we have no room for them.

---

*Gastric Juice.*—Dr. John C. Dalton, jr., of New York, has published in the American Journal of Medical Sciences, an account of some experiments upon the gastric juice.

He finds that the stomach, during the interval of digestion, contains only mucus, alkaline or neutral in its reaction. In about five minutes after the introduction of food, the acid fluid begins to run in drops, and in ten or fifteen minutes, flows freely, sometimes in a small stream, from the orifice of the fistula, and that four or five ounces can be collected from a dog of average size, in half an hour. It is first colorless, but afterwards ac quires a faint amber tinge. "Its specific gravity is 1010; its reaction always is acid; its viscidity is so slight as to be scarcely noticeable; it becomes turbid by boiling,* and by excess of alcohol; it is not troubled by nitric acid, nor by ferrocyanide of potassium."

Dr. D. objects to the method of attempting to procure *pure* gastric juice by introducing indigestible substances into the stomach. He says it uniformly fails. He uses firm pieces of meat, containing little blood, and having their fluids coagulated by a short preliminary boiling, and thinks that a sufficient length of time intervenes between the flow of the fluid and the commencement of digestion, to enable the experiment to obtain the juice perfectly pure. He says that neither albumen nor albuminose are found in the fluid thus obtained.

In regard to *pepsin,* he asserts that Lehman and Frerichs, are wrong in stating that its solution is not coagulated by heat, but as he gives us no account of the method he resorted to, to procure pure pepsin, and as the gastric juice he experimented on might be contaminated with albumen, we cannot regard his results as at all conclusive.

The gastric fluid acts only on albuminoid substances, which are converted into albuminose. The digestible ingredients of bread are all taken up in this form: cheese has its casein converted into albuminose and dissolved, while its oily portions are set free and float upon the surface as a yellow layer. Raw white of egg dissolves largely as albumen, while soft boiled white of egg and raw meat are principally converted into albuminose, a portion, however, remaining unaltered albumen.

He goes on to show that the gastric juice not only disintegrates, but actually dissolves the greater portion of the food, certain minute spots remaining unacted upon, and subsiding to the bottom of the test-tube. He expresses a doubt whether these refractory substances remain insoluble or are subsequently dissolved in the intestines. It is a little remarkable, that Dr. D. should have taken no notice of the numerous microscopical observations which prove that many morphological elements pass through the intestines unchanged.

The doctor makes a strange miscalculation in regard to the amount of gastric fluid necessary to perform the function of digestion. He finds that 50 grains of dried, or 250 of common meat will be dissolved by an ounce and a half of gastric juice, and then says, to dissolve a pound of ordinary meat, *"no less than sixteen pints of gastric juice will be necessary."* Any of our readers can make the calculation for himself. A troy pound contains 5760 grains; and if 250 grains are dissolved by an ounce and a half, it will only require 34½ ounces, or *a little over two pints* to dissolve the pound of emat.

Dr. Dalton goes on further to state, that amylaceous substances are not

* Other observers say this does not take place till after the acidity has been neutralized.

digested in the stomach, and insists that outside of the body, gastric juice totally prevents the conversion of starch into glucose. In this, he is opposed by all the eminent German chemists who have recently devoted so much time and attention to this subject. They are unanimously agreed that the gastric juice does not impede the action of saliva upon starch. He also denies that sugar is to be found, at any time, in the gastric juice. Now we well know that sometimes the starch will gradually disappear, as Dr. Dalton says, and yet no sugar be found, but this would seem to be because it is absorbed as fast as it is generated. In the majority of eases sugar is detected.

Bidder and Schmidt found that starch was immediately converted into sugar when the contents of the stomach were alkaline from excess of saliva, but that the change was greatly protracted when there was a decided acid reaction from the predominance of gastric juice. In all cases, however, the sugar was found. Frerichs, in fifty experiments upon men, quadrupeds and birds, always found sugar in the filtered gastric fluid. Gacubowitsch, also, has made very decisive experiments upon this point. He invariably found that all the starch was converted into sugar, in four or five hours, when the salivary glands remained intact, but that when the ducts were tied, no sugar nor even dextrine was to be discovered.

Dr. D. further states, that the presence of gastric juice interferes with Trommer's test for grape sugar, the solution taking a purple tinge instead of its usual blue color, and becoming yellow without letting fall a precipitate, after boiling.

---

*Dr. Charles R. Coffin,* a graduate of the Baltimore College of Dental Surgery, and for several years a highly esteemed practitioner of Portland, Me., has, within the last few months, located himself in Manchester, England, where, we are gratified to learn, his prospects of success are of the most flattering character. The principal object which Dr. C. had in view in leaving Portland was to avoid the severity of the protracted winters of that place, and being a man of high toned integrity and most agreeable manners, as well as a scientific and eminently skillful practitioner, he would scarely fail any where to secure public confidence and respect. We wish him the most abundant success in this new field of professional labor.

